# Challenges in diagnosing cryptococcosis among HIV-infected patients in southern Mozambique and opportunities for intervention in contexts of limited resources: A pre-implementation study

**DOI:** 10.1371/journal.pone.0340217

**Published:** 2026-02-05

**Authors:** José C. Langa, Troy D. Moon, Jahit Sacarlal, Mohsin Sidat

**Affiliations:** 1 Department of Microbiology, Faculty of Medicine, Eduardo Mondlane University, Maputo, Mozambique; 2 Department of Tropical Medicine and Infectious Diseases, Division of Pediatric Infectious Diseases, Tulane University Celia Scott Weatherhead School of Public Health and Tropical Medicine, New Orleans, Louisiana, United States of America; 3 Department of Community Health, Faculty of Medicine, Eduardo Mondlane University, Maputo, Mozambique; Mansoura University Faculty of Veterinary Medicine, EGYPT

## Abstract

**Introduction:**

Worldwide, cryptococcosis has increased due to HIV/AIDS, particularly in sub-Saharan Africa (SSA), where the mortality is high. Laboratory diagnosis for cryptococcosis among HIV-infected patients remains a challenge in most SSA countries. This study aimed to explore facilitators, barriers to re-implementing the India ink test, and logistical factors that could support the successful rollout of this alternative diagnostic method in southern Mozambique.

**Methods:**

We conducted a cross-sectional study using mixed methods approaches in six hospitals across southern Mozambique over three months. Clinicians and laboratory technicians were asked to complete a questionnaire aimed to capture sociodemographic data, as well as current practices and clinical approaches with respect to cryptococcal diagnosis and clinical management. Subsequently, individual in-depth interviews were conducted to understand current challenges in the diagnosis of cryptococcosis and to assess facilitators, barriers, and potential opportunities for interventions. Quantitative data were captured through REDCap and subsequently analyzed using SPSS statistical program, while qualitative data were analyzed manually using a combination of content and thematic analysis approaches.

**Results:**

A total of 98 healthcare providers (67 clinicians and 31 laboratory technicians) participated in the study. The mean age of the participants was 35 years. More than half were female (58.2%) and had at least five years of relevant work experience (53.1%). Challenges in diagnosing and managing cryptococcal disease were mainly related to 1) frequent shortages in the stocks of cryptococcal rapid antigen test, 2) contextual challenges in collecting cerebrospinal fluid samples, and 3) frequent shortages in the stocks of antifungal medications. In contrast, all study facilities had availability of microscopes, recognized the need for cryptococcal screening services, and had clinicians and laboratory technicians willing to collaborate for eventual roll-out of the test as routine practice.

**Conclusion:**

Frequent stock-outs of rapid diagnostic tests and challenges associated with performing lumbar punctures are barriers to routine screening for cryptococcosis. In contrast, we have identified the universal availability of microscopes and health workers’ recognition of the problem, as well as their willingness to collaborate, as potential facilitators to leverage for the successful reintroduction of India ink microscopy of urine samples as a low-cost laboratory test alternative.

## Introduction

Cryptococcosis is an important and life-threatening fungal infection [1]. In addition, Cryptococcus tops the list of priority opportunistic fungal pathogens due to its lethality among persons living with HIV (PLHIV) [2,3]. In 2020, the global annual incidence of cryptococcosis was estimated at 152,000 cases, resulting in roughly 112,000 cryptococcosis-related deaths [4]. More than 70% of cryptococcosis deaths occur in low- and middle-income countries (LMIC) [4,5].

In LMIC, HIV-associated cryptococcosis remains a major cause of hospital admissions and clinic visits, despite recent efforts to expand access to antiretroviral therapy (ART) [4,6]. This is particularly true among the most economically productive age groups [5,7,8]. It is estimated that approximately 75% of HIV-infected adults in LMIC do not have access to cryptococcal diagnostic tests, and suspected cases are generally treated empirically [5,6].

The 2021 prevalence of HIV infection in the adult population in Mozambique was estimated to be 12.5% [9]. Accurate data on the prevalence of cryptococcosis in the country are scarce, and in most cases, patients are diagnosed based on clinical suspicion alone. It is estimated that the number of new cryptococcosis cases in the country is around 18,600 cases per year (70.5 cases per 100,000 population) [5,10] and the prevalence of the disease among hospitalized HIV patients in the southern part of the country is roughly 5.3% [11].

Laboratory diagnosis is a high priority and essential for treating many diseases [12]. In resource-limited settings such as Mozambique, which is highly dependent on health-system resources made available by donor funding, the availability of laboratory technologies and other supplies is inconsistent. Mozambique has been unable to acquire and maintain sufficient quantities of the relatively expensive cryptococcal antigen tests (CrAg). In addition to cost, another challenge has been that these tests must be imported, and they have a relatively short shelf-life within which they can be used [13,14]. This makes purchasing in larger bulk quantities more difficult and has resulted in frequent supply chain shortages of the test in the country.

India ink microscopy has been used to diagnose cryptococcosis for more than a century [15]. Studies have shown that in persons infected with HIV, cryptococcus can be eliminated in the urine on average 22 days before the onset of cryptococcal meningitis symptoms [1,16]. In addition, urine samples can be easily collected and tested for cryptococcus at a relatively early stage of systemic infection, without need for invasive procedures such as lumbar puncture. India ink microscopy is simple, inexpensive, and easy to perform in the laboratory, however, the introduction and expansion of rapid diagnostic tests (RDT) for a variety of illnesses has contributed to an overall reduction in the use of microscopy over the last decade [15,17]. Furthermore, India ink microscopy, specifically, had fallen out of favor as a diagnostic test due to its relatively lower sensitivity (approximately 70–90%) and due to the introduction of newer RDT´s that were anticipated to be a superior alternative to India ink [15,17,18].

While CrAg has shown to have good sensitivity and specificity for diagnosing cryptococcosis, in some resource-limited settings, it has not reached its full potential, and its utilization for routine cryptococcal diagnosis remains a challenge due mainly to its availability. In Mozambique, this unrealized potential is due to challenges in maintaining appropriate stocks of the RDT, ensuring refrigeration conditions at health facility level, as well as ensuring quality control of the tests performed. As a result, clinicians often have to make treatment decisions based on clinical suspicion rather than confirmed laboratory diagnoses. In this context, we feel that re-introducing India ink microscopy for cryptococcal diagnosis, utilizing a non-invasively collected urine sample, is a low-cost, easily accessible laboratory test alternative to help countries like Mozambique overcome current challenges in diagnosing cryptococcus.

This study represents a baseline (pre-implementation) evaluation that was conducted as part of a larger multi-phase implementation science study designed to evaluate the re-introduction of India ink microscopy of urine for the diagnosis of cryptococcosis among HIV-infected adults within Mozambique´s primary healthcare facilities. Our goal is to identify barriers and facilitators that can guide and inform the re-introduction of India ink microscopy of urine as a low-cost laboratory diagnostic alternative for cryptococcosis.

## Materials and methods

### Study design and setting

We conducted a mixed-methods cross-sectional study for this analysis, utilizing both quantitative and qualitative approaches. The rationale for our pre-implementation study design is to gather evidence through which we can better understand current barriers and facilitators of cryptococcosis diagnosis and to explore best practices for the roll-out of India ink microscopy of urine as a low-cost and feasible alternative to CrAg testing. In this part of the study, we focus on gathering information from clinicians (physicians and nurses) involved in the direct care and treatment of adult HIV-infected patients, as well as from laboratory technicians working in the microbiology sections of our study site’s clinical laboratories.

A total of six health facilities were selected, by convenience, within Maputo and Gaza Provinces, in the southern part of Mozambique. The six health facilities selected are broken down by the following categories: One national referral hospital (Maputo Central Hospital); two general hospitals in Maputo City (Mavalane General Hospital and Jose Macamo General Hospital); two provincial hospitals (Matola Provincial Hospital in Maputo Province and Xai-Xai Provincial Hospital in Gaza Province); and one rural district hospital (Carmelo Hospital of Chókwè). These facilities were selected due to their established HIV care and treatment services, as well as their physical and human resource infrastructure.

### Recruitment of study participants

Clinicians involved in the direct provision of care to HIV infected adults and laboratory technicians involved in the processing of samples for the diagnosis of cryptococcosis in our study sites were approached for participation. Following a detailed explanation of the study’s goals and objectives, the selected healthcare providers were invited to participate and provide signed informed consent (S1 File). Recruitment of participants began on 1 December 2024 and ended on 28 February 2025.

### Ethics statement

The study was conducted in accordance with the Declaration of Helsinki and was reviewed and approved by the National Bioethics Committee for Health of Mozambique (Comité Nacional de Bioética para Saúde), (*Protocol Number: 48/CNBS/2023*). It has also received administrative approval from each of our six study health facilities. All healthcare providers were required to provide written informed consent before completing the questionnaire and/or participating in the interview.

### Data collection

Quantitative data were collected through a self-administered questionnaire that utilized close-ended questions designed to capture sociodemographic data of the participants, as well as current clinician practices regarding cryptococcosis diagnosis. Data were entered into a Research Electronic Data Capture (REDCap) database, a secure web-based platform for collecting, storing, and analyzing basic data from the desired population. In addition, the study team collected data through direct observation of laboratory infrastructure, equipment, and systems that could potentially be leveraged for cryptococcal diagnosis through India ink testing.

Additional qualitative data were collected through individual in-depth interviews conducted within a private setting at each of the different study health facilities. The purposive sampling method was employed to select clinicians involved in providing direct care to HIV-infected adults, and laboratory technicians involved in processing samples for cryptococcal diagnosis. Data saturation was reached when no new information or themes emerged from additional interviews [19].

All interviews, conducted in the Portuguese language, were audio-recorded and explored the following themes: routine screening and management of cryptococcosis, laboratory capacity, availability of laboratory test kits for cryptococcal diagnosis, and any challenges faced concerning the availability of cryptococcal laboratory tests and/or other resources needed for the diagnosis and treatment of patients with suspected cryptococcosis. Audio recordings were transcribed word-for-word into Portuguese. Selected quotes were subsequently translated into English for dissemination purposes by members of the study team who are fluent in both Portuguese and English. An interview guide/script was utilized, which allowed for deeper probing of topics raised through the self-administered questionnaires. We performed quality control by comparing the transcribed text with the audio recording to ensure that no information was omitted or added. We analyzed data using Word for Microsoft to organize data into themes and sub-themes.

### Data analysis

Quantitative data was analyzed using SPSS Software package version 25 (IBM, USA). Initial analysis was based on descriptive statistics using proportions for the responses from the surveys regarding demographic information and challenges and opportunities for reintroducing India ink microscopy of urine samples, and producing frequency tables and graphics for categorical variables. Measures of central tendency and dispersion were estimated for numerical variables.

We conducted qualitative analysis of transcribed interviews by organizing data into themes and sub-themes related to challenges in the diagnosis and management of the cryptococcal disease, and by capturing healthcare providers’ perspectives regarding the opportunities and importance of eventual reintroduction of India ink microscopy.

Qualitative and quantitative methods were triangulated to identify areas of convergent and divergent responses.

## Results

### Healthcare provider demographics

The study enrolled a total of 98 healthcare providers from six hospitals in southern Mozambique. Of the participants, a majority (72.3%) were aged 30 years or older, and more than half (58.2%) were female. A majority of participants had been in their current field of practice for more than five years (53.1%). Of the healthcare providers recruited, the vast majority were physicians, representing 92.5% of the clinicians involved in direct patient care and 63.3% of our overall total study participants. Laboratory technicians represented 31.6% of our study participants (Table 1).

**Table 1:** Demographic characteristics of study participants.

Variable (N = 98)	N (%)
Age	
< 30 years	26 (26.5%)
> 30 years	72 (72.3%)
Gender	
Female	57 (58.2%)
Male	41 (41.8%)
Years of Practice	
< 5 years	46 (46.9%)
≥ 5 years	52 (53.1%)
Health Care Provider category	
Physician	62 (63.3%)
Nurse	5 (5.1%)
Laboratory technicians	31 (31.6%)

From each of our study sites, both physicians and laboratory technicians agreed to participate in the study. While four eligible clinicians and two laboratory technicians declined to participate in the study, they did not agree to provide written informed consent. In contrast, nurses from just two study sites, Jose Macamo General Hospital (n = 4) and Maputo Central Hospital (n = 1), agreed to participate in the study. Actually, the Central, General, and provincial hospitals allocated a few nurses to care and treatment of adult HIV-infected patients, as they are the most specialized hospitals in the country, with more physicians than primary hospitals.

### Challenges in current practice related to diagnosis and management of cryptococcal disease

**Fig 1:**
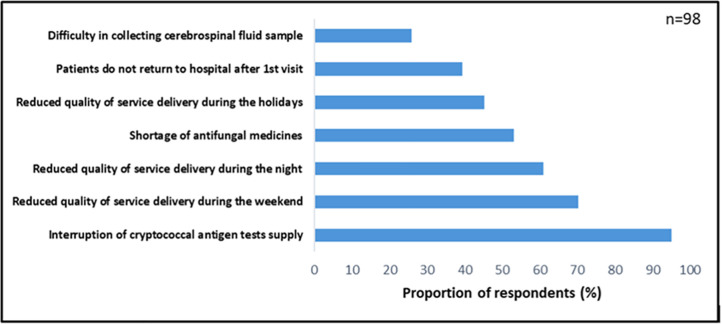
Challenges in diagnosing and managing cryptococcoses among HIV-infected patients in southern Mozambique.

Through the self-administered questionnaire, healthcare providers were questioned as to the challenges they currently experience with respect to the diagnosis and management of cryptococcal disease. *Stock outs of the CrAg rapid diagnostic test* were the answer provided by the largest proportion of participants (95%) (Fig 1). *Shortages in stocks of antifungal medications* were reported by 53.1% of participants, and *patients not returning for follow-up after the first visit* were reported by 39.4%. Other challenges reported included *shortages of staff during holiday periods, on weekends, and during night shifts*, compromising the quality of care and treatment services provided to patients during these off-hour rotations; and *difficulties in conducting the procedures to perform a lumbar puncture* to collect cerebrospinal fluid (CSF) for testing.

The challenges identified in current diagnostic practice were further explored through in-depth interviews with select healthcare providers (n = 16). The participants’ sentiments are described below with the inclusion of several illustrative English quotes (S2 File).

Thus, when asked about their experiences managing cryptococcosis among HIV-infected patients, half (4/8) of the clinicians interviewed described situations in which they had missed opportunities for timely diagnosis and treatment due to a lack of CrAg tests in their facility and due to a lack of other supplies needed to perform lumbar puncture.

*“I have missed opportunities to diagnose cryptococcal infection in my hospital because the laboratory did not have the cryptococcal antigen assay available…sometimes I felt frustrated because the hospital did not even have the tools to perform a lumbar puncture (Clinician #1)”.*

*“The lack of rapid tests, including resources such as catheters for collecting CSF is a major challenge for screening cryptococcosis in the hospital. Sometimes, I request the cryptococcal antigen test, and I don’t get a result back because the laboratory is facing interruption of the cryptococcal antigen test (Clinician #2)”.*

Further, when asked about the availability of antifungal medication at their health facilities, more than half (5/8) of the clinicians reported that due to frequent stockouts, they had missed opportunities for treating HIV-infected patients that they suspected of being cryptococcal positive.

*“I’m feeling frustrated because even if I get a positive [cryptococcal] result, I don’t have any way of treating it due to a lack of medications. I feel it’s the ‘golden rule’, to treat the patient at that moment because it could prevent them from developing meningitis, complications, or even death (Clinician #3).”*

Several clinicians (3/8) also described a sense of uncertainty in their skills to perform a lumbar puncture on these patients.

*“My lack of certain medical practices causes me uncertainty when I am faced with patients whose clinical situation requires a lumbar puncture. In this case, I don’t know what will happen to these patients in terms of future diagnosis. If a CSF sample is needed, I refer the patients suspected of having a cryptococcal disease to a specialist or colleague in the same department or to another healthcare facility that can perform lumbar punctures” (Clinician #4).*

*“My lack of skills in performing a lumbar puncture has been a major challenge when I have patients suspected of having cryptococcal disease. I perform the lumbar puncture but with the support of an experienced colleague.” (Clinician #5)”.*

### Current practices with regards to cryptococcal test request frequency

The clinician participants (n = 66), who are involved in the direct care and treatment of HIV-infected patients, were questioned as to the frequency that they requested cryptococcal testing when they had a patient with suspected cryptococcosis. Overall, slightly more than half (54.5%) responded that they “*always*” requested cryptococcal testing, while 37.9% responded “*sometimes*” and 7.6% responded “*never*”. We found no statistically significant differences in the frequency of cryptococcal test requests by the cadre of healthcare providers (p = 0.127) or by years of clinical practice experience (p = 0.272). However, statistically significant differences were found in the health facilities where the providers worked (p = 0.023) (Table 2).

**Table 2:** Frequency of cryptococcal diagnostic test requests.

	Frequency of Test Requests				
	Total	Always	Sometimes	Never	p-value
**Total responding**	66 (100.0%)	36 (54.5%)	25 (37.9%)	5 (7.6%)	
**Clinician category**					0.127
Physician	61 (92.4%)	35 (57.4%)	21 (34.4%)	5 (8.2%)	
Nurse	5 (7.6%)	1 (20.0%)	4 (80.0%)	0 (0.0%)	
**Years of clinical practice**					0.272
< 5 years	31 (47.0%)	15 (48.4%)	12 (38.7%)	4 (12.9%)	
≥ 5 years	35 (53.0%)	21 (60.0%)	13 (37.1%)	1 (2.9%)	
**Health facility**					0.023
MCH	15 (22.7%)	7 (46.7%)	8 (53.3%)	0 (0.0%)	
MGH	16 (24.2%)	7 (43.8%)	7 (43.8%)	2 (12.5%)	
JMGH	8 (12.1%)	2 (25.0%)	5 (62.5%)	1 (12.5%)	
MPH	9 (13.6%)	6 (66.7%)	3 (33.3%)	0 (0.0%)	
XPH	13 (19.7)	11 (84.6%)	2 (15.4%)	0 (0.0%)	
CHC	5 (7.6%)	3 (60.0%)	0 (0.0%)	2 (40.0%)	

MCH = Maputo Central Hospital; MGH = Mavalane General Hospital; JMGH = Jose Macamo General Hospital; MPH = Matola Provincial Hospital; XPH = Xai Xai Provincial Hospital; CHC = Carmelo Hospital of Chókwè.

Explorative in-depth interviews revealed the following (n = 16):

*“I sometimes request cryptococcal testing when I have a patient with suspected cryptococcosis, but during the weekend periods and at night, I feel that the laboratory sample might get lost due to the increased demand for hospital services and the heavy workload in the laboratory where only one laboratory technician is on duty (Clinician #6)”.*

*“I usually request a cryptococcosis test when I have a patient with suspected cryptococcosis as part of my routine, even though there is sometimes a delay in getting the results back from the laboratory, which forces me to resort to empirical treatment in this scenario. Furthermore, patients are not always able to have a lumbar puncture performed... if they have confusion or behavioral changes, so we have to sedate those patients to do a lumbar puncture or postpone taking the sample, and alternatively we have to resort to empirical treatment” (Clinician #7).”*

### Healthcare provider perspectives on the re-introduction of India ink microscopy of urine samples

**Fig 2:**
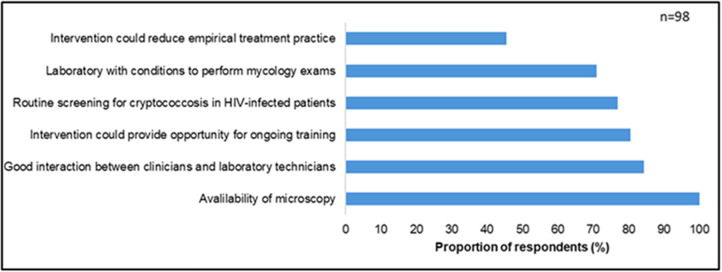
Perspectives on the reintroduction of India ink microscopy of urine samples to cryptococcosis diagnosis.

We questioned healthcare providers (n = 98) regarding their opinions and perspectives on the opportunities for reintroducing India ink microscopy, applied to urine, as an alternative tool to improve cryptococcosis diagnosis. Healthcare providers listed several “*opportunities*” that they felt would support the re-introduction of India ink testing. First, healthcare providers noted that all six study sites had microscopy services available, which could be directed towards India ink microscopy of urine. Next, 84.4% of participants described “*good interactions*” and/or “*interprofessional relationships*” between the clinicians and laboratory technicians of their facility, which would help facilitate the roll-out of this new intervention. A majority of healthcare providers (76.9%) reported that they felt the intervention had the potential to allow them to perform cryptococcal screening of HIV patients more routinely. Finally, 81% of laboratory technicians felt that an intervention to re-introduce India ink microscopy would be a good capacity-building exercise, as they lack regular in-service training opportunities (Fig 2).

While generally positive, our in-depth interviews revealed some uncertainties from the laboratory technicians interviewed:

*“If the clinician requested a cryptococcosis diagnosis through India ink microscopy…I have always supported him, but in my case, I feel that further training on microscopy is needed in order to perform the exams and enhance my confidence in using this technology” (Lab. Technician #1).*

*“Using microscopy to diagnose cryptococcosis on CSF is currently a challenge for me due to the lack of appropriate training; the last training in this technology was 5 years ago (Lab. Technician #2)”.*

*Regarding the new diagnosis, I am ready to collaborate, support the clinical team, and respond to their requests. I am satisfied that I have the opportunity to improve my performance in the microscopic diagnosis of cryptococcosis (Lab. Technician #3).*

## Discussion

In this study, we aimed to identify barriers and facilitators that can be used to guide and inform the re-introduction of India ink microscopy of urine as a low-cost laboratory diagnostic alternative for cryptococcus. Our study explores the perspectives of clinicians (physicians and nurses) involved in the direct care and treatment of adult HIV-infected patients, as well as from laboratory technicians working in the microbiology sections of our study site’s clinical laboratories. Their opinions and perspectives are useful in enhancing roll-out of the implementation strategy at each of the different study sites.

The study’s findings shed light on how barriers and facilitators may influence implementation, reflecting previously reported determinants in similar settings. Additionally, these findings highlight the need for a relatively low-cost and simple intervention to expand diagnostic opportunities for cryptococcosis in low-resource settings. Findings from this study also highlight the importance of maintaining the quality of care and treatment services provided during off-hour rotations. Considering that, the quality of health care delivered to patients has largely received little attention in LMICs [20] Additionally, a large proportion of patients with opportunistic infections and advanced HIV disease are rarely reported, mainly due to a lack of diagnostic and human resource capacity [15,21]. Studies highlight in resource-limited settings, in Mozambique and neighboring countries, challenges to the diagnosis of cryptococcosis, such as limited access to CrAg screening and a lack of lumbar puncture kits [15,22,23]. Additionally, surveillance systems for fungal infections are scarce [15]. CrAg has excellent performance (>99% sensitivity and specificity), is easy and quick to perform, and costs less than US$5 [15,17]. However, using these highly sensitive and specific tests in low-income countries remains a challenge [15,22]. In Mozambique, which is highly dependent on health-system resources made available by donor funding, the availability of laboratory technologies and other supplies is inconsistent. The findings of this study underscore the urgent need to expand point-of-care diagnostic testing for HIV-infected patients. We explored the opportunities to use India ink microscopy on urine samples as a routine alternative to CrAg for cryptococcosis diagnosis, particularly among HIV-infected patients, until CrAg access improves. Studies have shown that in persons infected with HIV, cryptococcus can be eliminated in the urine on average 22 days before the onset of cryptococcal meningitis symptoms [1,16]. Even with its lower reported sensitivity as a screening test, in the absence of good access to CrAg or other definitive diagnostics, we feel that routine testing of urine with India ink constitutes a “window” of opportunity for screening and subsequent initiation of treatment prior to the full establishment of cryptococcal meningitis manifestations. Utilizing India Ink microscopy on urine samples from individuals suspected of having cryptococcal infection offers a practical alternative to lumbar puncture, an invasive procedure, that can be a barrier to diagnosis. This relatively simple approach yields timely results and enables same-day treatment initiation.

These findings are consistent with previous research on point-of-care screening for cryptococcosis, which has shown that limited access to diagnostic tools hinders effective patient follow-up [17].

Some inexperienced laboratory technicians may mistake yeast cells for lymphocytes, especially if the yeast cells have a thin capsule. This limitation contributes to misdiagnosis or false negatives [24]. However, providing routine capacity building on India ink microscopy, supervision, and quality control may enhance the likelihood of accurate outcomes, reduce errors, and ensure competence for laboratory technicians [12,25,26].

The results also highlight the importance of strengthening Mozambique’s public health system to deliver services that improve the overall health and life expectancy of HIV-infected adults.

Additionally, the study findings identified good interprofessional relationships between the clinicians and laboratory technicians as a key facilitator for strengthening the intervention. These findings align with prior studies that highlight the importance of good interactions between stakeholders for the successful rollout of an intervention [27,28]. Previous studies have emphasized that strong collaboration and professional relationships between clinicians and laboratory staff are crucial for fostering trust in diagnostic tests, influencing clinicians’ test-ordering behavior and treatment decisions, increasing the use of laboratory diagnostics, and ultimately improving patient care. [12,28]. Hence, the laboratory professional should be the primary consultant to the clinician when the clinician is uncertain about a laboratory-specific issue [27].

Our pre-implementation evaluation highlighted not only the pressing challenges in diagnosing cryptococcosis, such as the frequent interruptions in the supply of cryptococcal antigen tests but also key opportunities that could support the rollout of our intervention, including the widespread availability of microscopy services at the study sites. These insights underscore the critical value of involving frontline healthcare providers who directly care for HIV/AIDS patients in shaping practical, context-sensitive diagnostic strategies before implementation begins [25].

Our study highlights the frequent stock-outs of antimicrobial medications, as reported by participants. This is particularly concerning given the high burden of infectious diseases in Mozambique, a low-income country with limited healthcare resources [29]. Additionally, delays in illness recognition at home and in seeking medical care are common [30]. These medication shortages contribute to prolonged delays in treatment initiation, increasing the risk of severe complications such as meningitis and even death [31].

We note that the healthcare providers enrolled in this study had at least five years of relevant work experience and were predominantly young. Additionally, they faced and recognized the real problem of diagnosing cryptococcosis among HIV-infected patients in southern Mozambique, within resource-limited settings.

Studies highlighted that perceptions of benefits and risks, as well as confidence in research, are significant factors in willingness to participate in research [32–34]. We feel that healthcare providers’ recognition of the need for routine cryptococcal diagnosis in HIV infected patients can lead to willingness to participate in this research. Additionally, the availability of microscopes in hospitals facilitates intervention, making it more cost-effective once the India ink is readily available and inexpensive.

Overall, the findings from this study inform implementation recommendations, focusing on an educational intervention that includes refresher training in microscopy to strengthen provider self-efficacy and diagnostic competence, thereby supporting the sustained use of the method.

### Limitations

The quantitative data for this analysis were collected through a self-administered questionnaire, which may have led participants to respond in a manner that reflects favorably on their health facility and its management, rather than providing fully honest answers. Additionally, the participants were selected via convenience sampling in only six facilities, which limits the generalizability of these results to all healthcare providers nationwide.

## Conclusion

In this baseline, pre-implementation evaluation, we identified both significant barriers and key facilitators that could influence the uptake and effectiveness of our proposed intervention to re-introduce India ink microscopy of urine as an alternative diagnostic method to increase opportunities for cryptococcosis diagnosis among HIV-infected patients. Stockouts of CrAg diagnostic tests disrupted the continuity and reliability of care, highlighting the need for our intervention. Additionally, staff shortages, especially during holidays and off-hours, further constrained the delivery of timely and effective services. Finally, healthcare providers’ hesitation in performing lumbar punctures highlights a need for more hands-on clinical training and confidence-building measures.

Despite these challenges, several contextual strengths are felt to facilitate implementation. Universal access to functional microscopes has the potential to provide a consistent diagnostic resource, and strong interpersonal relationships between clinicians and laboratory personnel promote teamwork and communication. Importantly, the India ink intervention is widely viewed as a valuable capacity-building opportunity, especially for laboratory staff seeking to refresh and strengthen their technical skills. Leveraging these existing assets while addressing supply chain and human resource gaps will be essential for improving long-term sustainability and impact.

## Supporting information

S1 File Consent form. (PDF)

S2 File Quotes in English-language version. (PDF)

## Abbreviations

AIDS: Acquired immunodeficiency syndrome
ART: Antiretroviral therapy
CHC: Carmelo Hospital of Chókwè
CM: Cryptococcal meningitis
CNBS: *Comité Nacional de Bioética para Saúde*
CrAg: Cryptococcal antigen tests
CSF: Cerebrospinal fluid
HIV: Human immunodeficiency virus
JMGH: Jose Macamo General Hospital
LMIC: Low- and middle-income countries
MCH: Maputo Central Hospital
MGH: Mavalane General Hospital
MPH: Matola Provincial Hospital
NHS: National Health Service
PLHIV: Persons living with HIV
RDT: Rapid diagnostic tests
REDCap: Research Electronic Data Capture
SSA: Sub-Saharan Africa
XPH: Xai-Xai Provincial Hospital.

## References

[1] Kambugu A, Meya DB, Rhein J, O’Brien M, Janoff EN, Ronald AR, et al. Outcomes of cryptococcal meningitis in Uganda before and after the availability of highly active antiretroviral therapy. Clin Infect Dis. 2008;46(11):1694–701. doi: 10.1086/587667 18433339 PMC2593910

[2] World Health Organization. WHO fungal priority pathogens list to guide research, development and public health action. 2022.

[3] Sati H, Alastruey-Izquierdo A, Perfect J, Govender NP, Harrison TS, Chiller T, et al. HIV and fungal priority pathogens. Lancet HIV. 2023;10(11):e750–4. doi: 10.1016/S2352-3018(23)00174-1 37827187 PMC7615271

[4] Rajasingham R, Govender NP, Jordan A, Loyse A, Shroufi A, Denning DW, et al. The global burden of HIV-associated cryptococcal infection in adults in 2020: a modelling analysis. Lancet Infect Dis. 2022;22(12):1748–55. doi: 10.1016/S1473-3099(22)00499-6 36049486 PMC9701154

[5] Rajasingham R, Smith RM, Park BJ, Jarvis JN, Govender NP, Chiller TM, et al. Global burden of disease of HIV-associated cryptococcal meningitis: an updated analysis. Lancet Infect Dis. 2017;17(8):873–81. doi: 10.1016/S1473-3099(17)30243-8 28483415 PMC5818156

[6] Link A, Okwir M, Nabongo B, Meya D, Iribarren S, Bohjanen P, et al. Delays in cryptococcal meningitis diagnosis and care: a mixed methods study in rural Uganda. Ann Glob Health. 2022;88(1):22. doi: 10.5334/aogh.3524 35415076 PMC8932357

[7] Limper AH, Adenis A, Le T, Harrison TS. Fungal infections in HIV/AIDS. Lancet Infect Dis. 2017;17(11):e334–43. doi: 10.1016/S1473-3099(17)30303-1 28774701

[8] WHO. Guidelines for the diagnosis, prevention and management of cryptococcal disease in HIV-infected adults, adolescents and childrens. 2018. 26110194

[9] INSIDA. Inquérito nacional sobre o impacto do HIV e SIDA em Moçambique. 2021.

[10] Sacarlal J, Denning DW. Estimated burden of serious fungal infections in Mozambique. J Fungi (Basel). 2018;4(3):75. doi: 10.3390/jof4030075 29937480 PMC6162438

[11] Deiss R, Loreti CV, Gutierrez AG, Filipe E, Tatia M, Issufo S, et al. High burden of cryptococcal antigenemia and meningitis among patients presenting at an emergency department in Maputo, Mozambique. PLoS One. 2021;16(4):e0250195. doi: 10.1371/journal.pone.0250195 33901215 PMC8075188

[12] Petti CA, Polage CR, Quinn TC, Ronald AR, Sande MA. Laboratory medicine in Africa: a barrier to effective health care. Clin Infect Dis. 2006;42(3):377–82. doi: 10.1086/499363 16392084

[13] Rönninger SK, Garbe JHO. Import testing turned into an unnecessary limitation of patient access to medicines as risks are managed effectively. Pharm Policy Law. 2016;18(1–4):141–56. doi: 10.3233/ppl-160439

[14] The fundamentals of stability testing. Weymouth, England: Micelle Press; 1992.

[15] Lakoh S, Kamudumuli PS, Penney ROS, Haumba SM, Jarvis JN, Hassan AJ, et al. Diagnostic capacity for invasive fungal infections in advanced HIV disease in Africa: a continent-wide survey. Lancet Infect Dis. 2023;23(5):598–608. doi: 10.1016/S1473-3099(22)00656-9 36565714

[16] Jarvis JN, Percival A, Bauman S, Pelfrey J, Meintjes G, Williams GN, et al. Evaluation of a novel point-of-care cryptococcal antigen test on serum, plasma, and urine from patients with HIV-associated cryptococcal meningitis. Clin Infect Dis. 2011;53(10):1019–23. doi: 10.1093/cid/cir613 21940419 PMC3193830

[17] Rajasingham R, Wake RM, Beyene T, Katende A, Letang E, Boulware DR. Cryptococcal meningitis diagnostics and screening in the era of point-of-care laboratory testing. J Clin Microbiol. 2019;57(1):e01238-18. doi: 10.1128/JCM.01238-18 30257903 PMC6322457

[18] Boulware DR, Rolfes MA, Rajasingham R, von Hohenberg M, Qin Z, Taseera K, et al. Multisite validation of cryptococcal antigen lateral flow assay and quantification by laser thermal contrast. Emerg Infect Dis. 2014;20(1):45–53. doi: 10.3201/eid2001.130906 24378231 PMC3884728

[19] Guest G, Bunce A, Johnson L. How many interviews are enough? Field Methods. 2006;18(1):59–82. doi: 10.1177/1525822x05279903

[20] Titi-Ofei R, Osei-Afriyie D, Karamagi H. Monitoring quality of care in the WHO Africa region-A study design for measurement and tracking, towards UHC attainment. Glob Health Action. 2021;14(1):1939493. doi: 10.1080/16549716.2021.1939493 34320908 PMC8330734

[21] Bongomin F, Gago S, Oladele RO, Denning DW. Global and multi-national prevalence of fungal diseases-estimate precision. J Fungi (Basel). 2017;3(4):57. doi: 10.3390/jof3040057 29371573 PMC5753159

[22] Falconer J, Mmotsa TM, Govender NP, Jarvis JN. Diagnosis of cryptococcal meningitis in people living with HIV in low-income countries: barriers and strategies. Expert Rev Anti Infect Ther. 2025;23(10):893–905. doi: 10.1080/14787210.2025.2554999 40874873

[23] Saylor D, Elafros M, Bearden D, Dallah I, Mathews M, Muchanga G, et al. Patient, provider, and health systems factors leading to lumbar puncture nonperformance in Zambia: a qualitative investigation of the “tap gap”. Am J Trop Med Hyg. 2023;108(5):1052–62. doi: 10.4269/ajtmh.22-0699 36972691 PMC10160901

[24] Oladele R, Bongomin F, Gago S, Denning D. HIV-associated cryptococcal disease in resource-limited settings: a case for “prevention is better than cure”? JoF. 2017;3(4):67. doi: 10.3390/jof3040067 29371581 PMC5753169

[25] Rogers HL, Pablo Hernando S, Núñez - Fernández S, Sanchez A, Martos C, Moreno M, et al. Barriers and facilitators in the implementation of an evidence-based health promotion intervention in a primary care setting: a qualitative study. J Health Organ Manag. 2021;35:349–67. doi: 10.1108/JHOM-12-2020-0512 PMC9136863 34464035

[26] Abimiku AG, Institute of Human Virology, University of Maryland School of Medicine PEPFAR Program (AIDS Care Treatment in Nigeria [ACTION]). Building laboratory infrastructure to support scale-up of HIV/AIDS treatment, care, and prevention: in-country experience. Am J Clin Pathol. 2009;131(6):875–86. doi: 10.1309/AJCPELMG6GX6RQSM 19461097

[27] Armstrong E, Joutsi-Korhonen L, Lassila R. Interaction between clinic and laboratory. Thromb Res. 2011;127 Suppl 2:S2-4. doi: 10.1016/S0049-3848(10)70146-8 21193109

[28] Tuijn CJ, Msoka E, Mushi DL, Sumari-de Boer M, Chilongola J, Van den Broek A. The interface between clinicians and laboratory staff: a field study in northern Tanzania. Afr J Lab Med. 2014;3(1). doi: 10.4102/ajlm.v3i1.126 PMC5637763 29043178

[29] Mabey D, Peeling RW, Ustianowski A, Perkins MD. Diagnostics for the developing world. Nat Rev Microbiol. 2004;2(3):231–40. doi: 10.1038/nrmicro841 15083158

[30] Garcia Gomez E, Igunza KA, Madewell ZJ, Akelo V, Onyango D, El Arifeen S, et al. Identifying delays in healthcare seeking and provision: The Three Delays-in-Healthcare and mortality among infants and children aged 1-59 months. PLOS Glob Public Health. 2024;4(2):e0002494. doi: 10.1371/journal.pgph.0002494 38329969 PMC10852234

[31] Saifodine A, Gudo PS, Sidat M, Black J. Patient and health system delay among patients with pulmonary tuberculosis in Beira city, Mozambique. BMC Public Health. 2013;13:559. doi: 10.1186/1471-2458-13-559 24499197 PMC3680113

[32] King TL, Tan SH, Tan SSN, Lai WH, Bujang MA, Voon PJ. Survey of willingness to participate in clinical trials and influencing factors among cancer and non-cancer patients. Sci Rep. 2025;15(1):1626. doi: 10.1038/s41598-024-83626-7 39794348 PMC11723972

[33] Sellars B, Garza MA, Fryer CS, Thomas SB. Utilization of health care services and willingness to participate in future medical research: the role of race and social support. J Natl Med Assoc. 2010;102(9):776–86. doi: 10.1016/s0027-9684(15)30674-x 20922921 PMC3110686

[34] Platt J, Raj M, Büyüktür AG, Trinidad MG, Olopade O, Ackerman MS, et al. Willingness to participate in health information networks with diverse data use: evaluating public perspectives. eGEMs. 2019;7(1):33. doi: 10.5334/egems.288 31367650 PMC6659576

