# Supplementary material for: Challenges in diagnosing cryptococcosis among HIV-infected patients in southern Mozambique and opportunities for intervention in contexts of limited resources: A pre-implementation study
Source: PLoS One. 2026 Feb 5;21(2):e0340217. doi: 10.1371/journal.pone.0340217 (PMC12875571; doi:10.1371/journal.pone.0340217)
Supplement: S1 File — (PDF) [file pone.0340217.s001.pdf]

CONSENT FORM

(HCPs)

Participant code_________

Having been invited to participate in the study**:** *"* Challenges in diagnosing cryptococcosis among HIV-infected patients in southern Mozambique and opportunities for intervention in contexts of limited resources: A pre-implementation study ."

I declare that:

1. I have been satisfactorily informed that the purpose of this research is to explore facilitators, barriers to re-implementing the India ink test, and logistical factors that could support the successful rollout of this alternative diagnostic method in southern Mozambique.
2. I have been informed of the nature of my participation in this research and the associated risks and benefits.
3. I understand that I will not receive any material or monetary compensation for participating in the study.
4. I have been duly informed of my right to withdraw from the study at any time without prejudice
5. I understand that my participation is voluntary, will be confidential, and that sections from the interview may be included in the publications to come from this research, with the understanding that the quotations will be anonymous.
6. I understand that I will participate in a 25-minute interview with audio recording.
7. I also understand that if I have any questions, I can ask them by contacting the principal investigator in this study at any time by calling +258 847301265.
8. Or if I have any questions about my rights as a participant in this research, or if I feel that I have not been treated appropriately, I can contact the *Comité Nacional de Bioética para a Saúde* (CNBS) of the Ministry of Health by calling +258 824066350, located at Av. Eduardo Mondlane/Salvador Allende - Maputo, 2nd floor.
9. I understand that I will be given a copy of this signed Consent Form.

Participant signature — Date and time

Participant's name (in capital letters)

Signature of the person who provided the consent explanation — Date and time

Name (in capital letters) of the person who explained the consent form
