# Supplementary material for: Challenges in diagnosing cryptococcosis among HIV-infected patients in southern Mozambique and opportunities for intervention in contexts of limited resources: A pre-implementation study
Source: PLoS One. 2026 Feb 5;21(2):e0340217. doi: 10.1371/journal.pone.0340217 (PMC12875571; doi:10.1371/journal.pone.0340217)
Supplement: S2 File — (PDF) [file pone.0340217.s002.pdf]

S2_File: Quotes in English-language version

Sub-theme	Question asked	Code	Quotation	Participant code	Subgroup
Challenges in current practice related to the diagnosis and management of cryptococcal disease	What is your experience in diagnosing cryptococcosis in HIV-infected patients?	*Diagnosis*	*"I have missed opportunities to diagnose cryptococcal infection in my hospital because the laboratory did not have the cryptococcal antigen assay available…sometimes I felt frustrated because the hospital did not even have the tools to perform a lumbar puncture."*	1C	Clinician
			"The lack of rapid tests, including resources such as catheters for collecting CSF is a major challenge for screening cryptococcosis in the hospital. Sometimes, I request the cryptococcal antigen test, and I don't get a result back because the laboratory is facing an interruption in the cryptococcal antigen test (Clinician).	2C	Clinician
	What do you think about the availability of antifungal medication at your health facility?	*Availability of antifungal medication*	*"I'm feeling frustrated because even if I get a positive [cryptococcal] result, I don't have any way of treating it due to a lack of medications. I feel it's the 'golden rule', to treat the patient at that moment because it could prevent them from developing meningitis, complications, or even death ".*	3C	Clinician
	How do you feel when you are required to perform a lumbar puncture on patients suspected of having cryptococcosis?	*Skills to perform lumbar puncture*	*"My lack of certain medical practices causes me uncertainty when I am faced with patients whose clinical situation requires a lumbar puncture. In this case, I don't know what will happen to these patients in terms of future diagnosis. If a CSF sample is needed, I refer the patients suspected of having a cryptococcal disease to a specialist or colleague in the same department or to another healthcare facility that can perform lumbar punctures".*	4C	Clinician
			"My lack of skills in performing a lumbar puncture has been a major challenge when I have patients suspected of having cryptococcal disease. I perform the lumbar puncture, but with the support of an experienced colleague".	5C	Clinician

Current practices with regards to cryptococcal test request frequency	How often do you request a cryptococcal diagnostic test when you have a patient with suspected cryptococcosis?	*Adherence to diagnostic tests*	*“I sometimes request cryptococcal testing when I have a patient with suspected cryptococcosis, but during the weekend periods and at night, I feel that the laboratory sample might get lost due to the increased demand for hospital services and the heavy workload in the laboratory where only one laboratory technician is on duty ”.*	6C	Clinician
			“I usually request a cryptococcosis test when I have a patient with suspected cryptococcosis as part of my routine, even though there is sometimes a delay in getting the results back from the laboratory, which forces me to resort to empirical treatment in this scenario. Furthermore, patients are not always able to have a lumbar puncture performed... if they have confusion or behavioral changes, so we have to sedate those patients to do a lumbar puncture or postpone taking the sample, and alternatively we have to resort to empirical treatment”.	7C	Clinician
Healthcare provider perspectives on the re-introduction of India ink testing of urine samples	What do you think about re-introducing India ink staining applied to urine as an intervention to increase opportunities for cryptococcosis diagnosis?	Uncertainties and expectantly	*“If the clinician requested a cryptococcosis diagnosis through India ink microscopy…I have always supported him, but in my case, I feel that further training on microscopy is needed in order to perform the exams and enhance my confidence in using this technology”.*	1TL	Laboratory Technician
			“Using microscopy to diagnose cryptococcosis on CSF is currently a challenge for me due to the lack of appropriate training; the last training in this technology was 5 years ago ”.	2TL	Laboratory Technician
			“I am pleased and encouraged... to know that urine, being an easily obtained sample, can be an alternative for maintaining routine diagnosis of cryptococcosis in HIV-infected patients using India ink, until the other missing tests are restored.”	8C	Clinician
			“Regarding the new diagnosis, I am ready to collaborate, support the clinical team, and respond to their requests. I am satisfied that I have the opportunity to improve my performance in the microscopic diagnosis of cryptococcosis ”.	3TL	Laboratory Technician
